# Studying the effects of booster shots and antibody responses to the SARS-CoV-2 vaccination over time in health personnel

**DOI:** 10.3389/fcimb.2023.1138631

**Published:** 2023-03-16

**Authors:** Jingjing Wu, Hanyou Mu, Xiaowan Pan, Wenzheng Guo

**Affiliations:** ^1^ Department of Laboratory Medicine, Shanghai East Hospital, School of Medicine, Tongji University, Shanghai, China; ^2^ Clinical Laboratory, Yiwu Maternal and Child Health Hospital, Jinhua, Zhejiang, China; ^3^ ETHealthCare, Shanghai, China

**Keywords:** antibody, booster shots, SARS-CoV-2, BBIBP-CorV, vaccine

## Abstract

**Background:**

With the emergence of mutant versions that lead to continual spreading and recurrent infections of SARS-CoV-2, the COVID-19 vaccines can assist protection for high risk groups, particularly health workers. Even while booster shots have been widely used, longitude studies on immune responses in healthy subjects are uncommon.

**Methods:**

Eighty-five healthcare workers who received the BBIBP-CorV vaccine were prospectively enrolled and monitored for up to ten months. Automated Pylon immunoassays were used to quantify total anti-SARS-CoV2 antibody levels (TAb), surrogate neutralization antibody levels (NAb), and antibody avidities over the course of the follow-up. Additionally, hematology analyses were performed.

**Results:**

Pylon antibody testing revealed that every participant tested negative at the beginning, and 88.2% of them tested positive about 14 days after receiving their second dosage. The TAb levels and NAb levels peaked in 76.5% and 88.2% of the subjects, respectively, at the same time. Age was connected with the peak antibody levels, but not with gender, BMI, or baseline hematological factors. The positive rates and the antibody levels had already started to decline three months following the second injection. The antibody levels and avidities quickly increased following the booster doses to levels that were considerably greater than the peak antibody responses before to the booster shots. Hematology testing revealed no safety concerns with immunizations.

**Conclusion:**

In healthy workers, the two doses of BBIBP-CorV were able to induce humoral immunity; however, 3 months following vaccination, the antibody levels started to decline. The BBIBP-CorV booster injections increase both the quantity and quality of antibodies, which gave support for utilizing booster doses to prolong the duration of the vaccine’s protective effects.

## Introduction

Since its initial discovery in December 2019, the coronavirus disease (COVID-19) has been a global pandemic (Zhou et al., 2020). SARS-CoV-2, the causative agent of the disease, is a virus of the genus Betacoronavirus closely related to the SARS-CoV which was discovered in 2002 and also caused severe acute respiratory syndromes (Wu et al., 2020). Due to COVID-19’s high mortality rate during the start of the pandemic, there have been widespread attempts from many different disciplines to treat and control the disease. Among them, vaccines were the main strategy taken to stop the virus’s spread and stop COVID-19 victims from developing severe cases and dying. Over thirty vaccines have been approved and used by countries around the world, which were based on different vaccine platforms and technologies including mRNA vaccines, adenoviral vector-based vaccines, recombinant protein vaccines and inactivated virus vaccines (Piccaluga et al., 2022). The Sinopharm inactivated viral vaccine BBIBP-CorV was widely utilized in mainland China. Observations of the immunogenicity of BBIBP-CorV and its protectiveness against infection, severe illness development, and mortality by these vaccines were made after two doses of BBIBP-CorV in pre-marketing clinical trials (Al Kaabi et al., 2021; Xia et al., 2021; Xia et al., 2022) and real-world studies (Zhang et al., 2021; Ismail AlHosani et al., 2022). However, a booster shot has been recommended to increase protection against the ongoing spread and recurring infections caused by SARS-CoV-2. The results of the longitude studies on the antibody responses in healthy subjects from the baseline to the booster doses show that the booster doses are efficacious. In this study, we assessed the maturation of antibody avidity following first-time BBIBP-CorV vaccination and reported the kinetics of antibody responses in healthcare professionals who received three doses of the vaccine over a period of 10 months.

## Materials and methods

### Study design and participants

Eighty-five healthcare workers eligible to receive the inactivated SARS-COV-2 vaccine (BBIBP-CorV, Sinopharm, Beijing, China) were prospectively enrolled. All the participants completed vaccinations by administering two doses of the vaccine 1 month apart. Blood samples were collected on the day of the first and second shots of the vaccines, as well as one month, three months and 6 months after the second shot from each participant. Forty-five out of the eighty-five participants had the third shots of the vaccine, the booster shots, approximately 10 months after the first shots. For those forty-five participants, additional blood samples were also collected approximately one month after the booster shots. EDTA anti-coagulated whole blood samples were used for analysis by a hematology analyzer and a flow cytometry within 24 hours. Serum samples were prepared by centrifugation and stored at -80°C for the measurements of antibodies in batches. The study was approved by the institutional ethical committee and complied with the Declaration of Helsinki. Written informed consent were obtained from each participant.

### Measurements of antibodies by Pylon SARS-CoV-2 Total Antibody assays and Pylon Surrogate Neutralization Antibody assays

The anti-SARS-CoV-2 Abs in the serum samples were assessed by Pylon TAb assays and Pylon NAb assays, which were probe-based fluorescent immunoassays performed on the Pylon 3D analyzer (ET Healthcare, China). The details of both assays were described previously (Yang et al., 2021). In brief, the Pylon TAb assay measured the total Abs bound to the RBD of the spike protein of SARS-CoV-2, while the Pylon NAb assays measured the overall inhibition of bindings between the RBD and the ACE2 protein by Abs in serum. The Pylon TAb assays returned semi-quantitative results as strong positive, positive, weak positive and negative together numbers in relative fluorescent units (RFU) representing fluorescence signals read from the samples. Meanwhile, the results of the Pylon NAb assays were reported as positive, strong positive or negative with the percentages of inhibition (% inhibition) on RBD-ACE2 binding by Abs in the samples. Both the RFU in the Pylon TAb assays and the % inhibition of the Pylon NAb assays were positively associated with the antibody titers or the neutralization activities (Yang et al., 2021).

### Measurements of the antibody avidities

The avidities of Abs reflected affinity maturation and multivalent binding development of Abs. In this study, they were measured by the relative dissociation rates (dRs) of the Abs-RBD bindings using the reagents of Pylon TAb assays. The slower the dRs were, the higher the avidities were. The method was reported previously and shown to correlate well with a Bio-Layer Interferometry avidity assay (Racine-Brzostek et al., 2021). To measure the dRs, the serum sample was titrated to an optimal concentration and an initial fluorescent signal of the Abs-RBD bindings was obtained when the Abs-RBD immune complexes were formed on the probe of the test as in a Pylon TAb assay. Then the immune complexes were incubated in PBS with Tween-20 (pH7.4) (PBST) for 30 seconds and the fluorescent signal was read again. The incubation-reading cycles were repeated several times to obtain a dissociation curve where the incubation lengths were plotted as the x-axis and the decreasing signals as the y-axis for calculating the dRs.

### Hematology analysis and measurements of CRP

Hematology analysis was performed using Mindray BC-6000 according to the standard operating procedure of the clinical laboratory in Shanghai East Hospital (south Branch). In brief, EDTA-K2 anti-coagulated whole blood was collected and hematology analysis was automatically performed within 30 minutes of collection. After hematology analysis, the above sample was used to measure the level of CRP (C-reactive protein) by Ottoman automatic real-time detection analyzer (UPPER Biotech Pharma CO., LTD, Shanghai, China).

### Flow cytometry

To determine the effects of vaccination on lymphocyte’s absolute number, the cell counts of T lymphocytes, B lymphocytes, NK cells and the subgroups (CD4+ or CD 8+) of T cells were determined by BD Multitest 6-color TBNK reagents on a BD FACSLyric flow cytometry (BD Bioscience, USA) according to the package insert. In brief, well-mixed whole blood samples were incubated with the 6-color TBNK reagents for 15 minutes in dark at 20-25°C in BD Trucount™ tubes before the red cells in the samples were lysed by adding the BD lysing solution. Then the samples were loaded and cell counts were measured on the flow cytometry and analyzed by BD FACSuite software.

### Statistical analysis

Categorical variables are expressed as percentages. Continuous variables are expressed as the mean ± SD or median (interquartile) according to normal or non-normal distribution. Statistical differences between groups were compared using the χ2 tests for categorical variables, the Kruskal-Wallis tests for non-normally distributed continuous variables and the One-way ANOVA for normally distributed variables. T-tests and Wilcoxon tests were used to compare the paired results. All the statistical analysis was performed using MedCalc version 20.009.

## Results

### Participants demographics

Eighty-five healthcare professionals participated in the study and their characteristics were summarized in **Table 1**. All of them had completed two doses of the BBIBP-CorV vaccine administered according to the package inserts. They were all followed up till six months after the second dose or seven months after the first dose. Forty-five participants had third shots of the vaccine as booster shot and were followed up till about one month after the third shot or 10 months after the first shots.

**Table 1:** Participant characteristics.

	Participants
Number	85
Gender, Male (%)	40(47.06%)
Height (cm), Mean (SD)	167.12 (8.99)
Weight (kg), Median (IQR)	59 (53.50-73.75)
Age (years), Median (IQR)	32 (26.75-44)
BMI (kg/m²), Mean (SD)	22.4 (2.67)

## Dynamics of antibody responses

### Seroconversion

**Figure 1:**
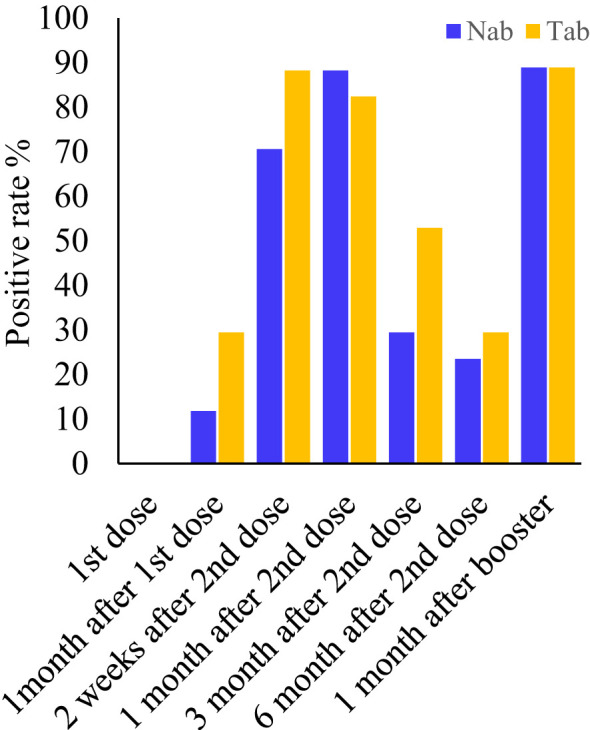
Positive rates of the Pylon TAb assays and the Pylon NAb assays at each time point. Time point (TP) 1-7: at the injection of the first dose, at the injection of the second dose (or ~1 month after the first dose), two weeks after second dose, 1 month after the second dose, 3 months after the second dose, 6 months after the second dose and 2 weeks-1 month after the third dose (or ~10 months after the first dose).

Serum samples collected at all seven-time points during the study period were measured by Pylon TAb assays and Pylon NAb assays. The positive rates at each time point were provided in **Table 2** and **Figure 1**. None of the eighty-five participants tested positive by either assay at the baseline when the first dose of vaccination was administered (Time Point 1, TP1), indicating no recent infections. The seroconversion was observed when the second dose of vaccination were administered 4 weeks after the first dose (TP2). Positive rates increased significantly two weeks after the second dose (TP3) with 70.6% and 88.2% of participants testing positive by Pylon TAb assays and Pylon NAb assays respectively. Similar overall positive rates (88.2% for Pylon TAb assays and 82.4% for Pylon NAb assays) were observed 1 month after the second dose (TP4). However, the positive rates decreased to 29.4% for Pylon TAb assays and 52.9% for Pylon NAb assays at 3 months post the second shots (TP5), and further to 23.5% for Pylon TAb assays and 29.4% for Pylon NAb assays 6 months post the second doses (TP6). Five participants remained negative from TP1 to TP6. Forty-five out of eighty-five participants had the third dose approximately 9 months after their first doses. All but five of them (88.89%) tested positive by both Pylon TAb assays and Pylon NAb assays 2 weeks-1 months after the booster dose (TP7). Among these forty-five participants, five of them tested negative by both assays at TP7, who had positive results of both Pylon TAb assays and Pylon NAb assays at TP3 and TP4. 

**Table 2:** Positivity rates of Pylon TAb assays and Pylon NAb assays during 10 months’ follow-up of the participants.

Time Point		n	Positive rates	
			TAb	NAb
1	first dose	85	0%	0%
2	second dose (month after first dose)	85	29.4%	11.8%
3	two weeks after second dose	85	88.2%	70.6%
4	1 month after second dose	85	82.4%	88.2%
5	3 months after second dose	85	52.9%	29.4%
6	6 months after second dose	85	29.4%	23.5%
7	two weeks – 1months after third dose (~10 months after first dose)	45	88.9%	88.9%

### Antibody kinetics

**Figure 2:**
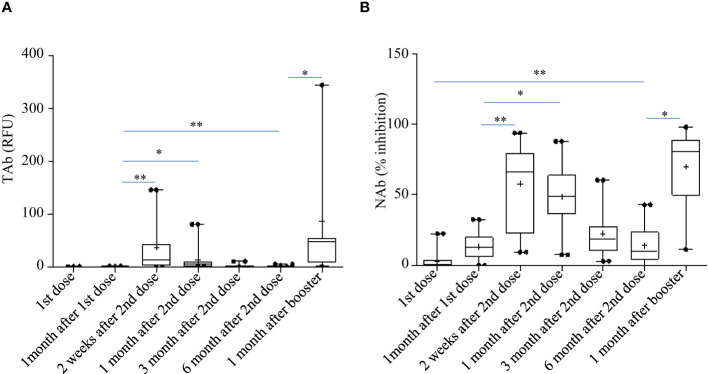
Total anti-RBD antibody levels measured by the Pylon TAb assays **(A)** and the surrogate neutralization abilities measured by the Pylon NAb assays **(B)** during the study. The horizontal lines indicated the medians and the error bards indicted the IQRs. Only the samples of 45 participants were available at TP7. * p<0.05 ** p<0.01.

Pylon TAb assays and Pylon NAb assays provided quantitative measurements of total anti-RBD antibody levels and surrogate neutralization abilities respectively. Both the total anti-RBD antibody levels and the surrogate neutralization abilities gradually increased after vaccination (**Figure 2**). At the second shot of the vaccines, the total antibody levels were slightly but significantly higher than the baseline (Time Point, median [IQR]: TP2, 0.29 [0.13-0.76] RFU vs TP1, 0.14 [0.14-0.19] RFU). The total antibody levels increased further around two weeks after the second dose (TP3 13.29 [2.037 - 44.645] RFU), but started to decrease around 1 month after the second dose (TP4, 5.29 [1.13 - 9.08] RFU). However, at both TP3 and TP4, the TAb levels were significantly higher than the previous (TP1 and TP2) (**Figure 2A**). Similarly, the NAb levels at two weeks (TP3, 66% [21.5%-81.2%]) and 1 month post the second doses (TP4, 49% [35.2%-65%]) were both higher (**Figure 2B**).

**Figure 3:**
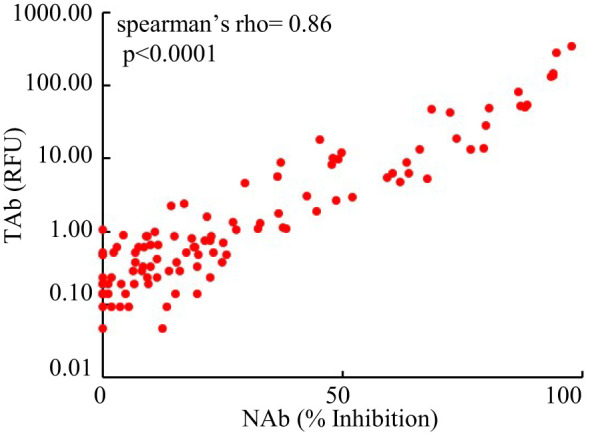
Comparison of the results of the Pylon TAb assays and the Pylon NAb assays (spearman’s rho= 0.86, p<0.0001).

However, the antibody responses decreased significantly by 3 months after the second shots (TAb: TP5, 0.905 [0.62-1.83] RFU; Nab: TP4, 17% [10.5%-31.5%]) and further around 6 months after the second shots (TAb: TP6, 0.48 [0.418-0.87] RFU; Nab: TP6 10% [3.5%-23.8%]) in both assays. Yet, around 6 months after the second shots (TP6) the antibody responses were still significantly higher than those at the baseline (TP6 vs TP1: TAb *p* =0.0003, Wilcoxon test; NAb: *p* =0.002, Wilcoxon test) and at the second doses (TP6 vs TP2: TAb *p* =0.0003, Wilcoxon test; NAb: *p* =0.8116, paired t-test) (**Figures 2A, B**). Forty-five participants had booster shots around 9 months after their first shots. In a month time (TP7), their antibody responses quickly increased again (TAb: TP7, 48.52 [11.0-109.0] RFU; Nab: TP4, 81% [49.5%-89.5%]) (**Figures 2A, B**). Thus, the dynamic changes of antibody responses from the baseline to six months after the second shots determined by the two assays were similar. The results of the two assays were well correlated (spearman’s rho= 0.860, *p <*0.0001, **Figure 3**).

**Figure 4:**
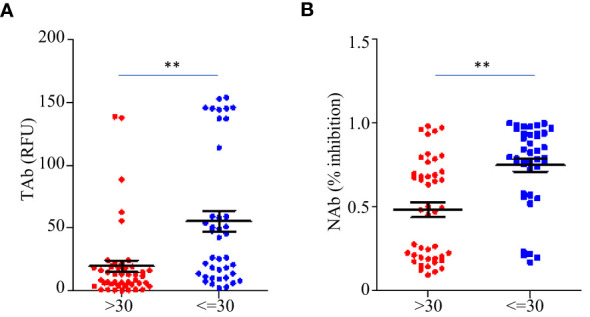
Comparison of the peak antibody responses TAb **(A)** and NAb **(B)** between participants younger than 30 years old (n=40) and older (n=45) before the booster shots. The horizontal lines indicated the medians and the error bards indicted the IQRs. ** p<0.01, Mann-Whitney test.

Before the booster shots (between TP1 and TP6), the total anti-RBD antibody levels (TAb) reached peaks around two weeks after the second dose (TP3) in sixty-five out of eighty-five participants (76.5%), while in seventy-five out of eighty-five participants (88.2%) the maximum neutralization abilities (NAb) were achieved around the same time. The peak antibody responses negatively correlated to ages (TAb vs Age: Spearman’s rho=-0.674 *p* =0.003, NAb vs Age: Spearman’s rho=-0.7 *p* =0.0019), but they were not associated with weight, BMI or gender. The peak responses in the participants younger than 30 years old were higher (**Figures 4A, B**). After the booster shots, forty out of the forty-five participants showed even higher antibody responses than their peak responses before the booster shots.

### Effects of the booster shots on the antibody responses

**Figure 5:**
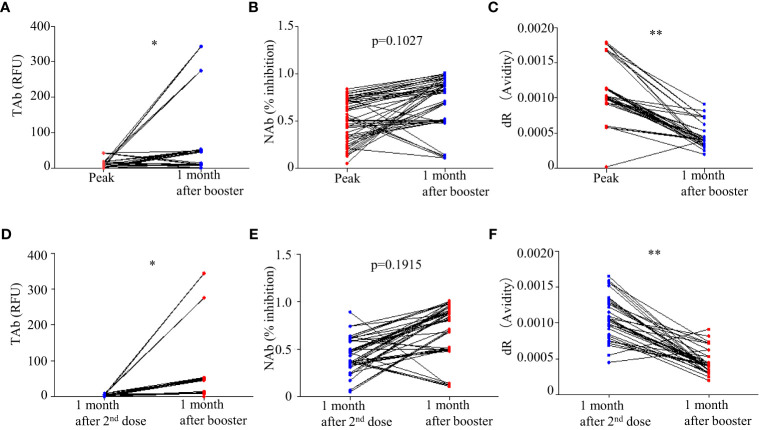
Comparison of antibody responses after the third doses (TP 7) with the peak responses after the second doses: **(A)** TAb (p <0.05, Wilcoxon test), **(B)** NAb (p = 0.1027, paired t-test), and **(C)** antibody avidity (p < 0.0001, paired t-test). **(D–F)** Comparison of antibody responses after the third doses (TP 7) with the second shots (TP4). * p<0.05 ** p<0.01.

Forty-five participants had the third dose of vaccination to boost the antibody responses 9 months after the first doses. As shown in **Figure 2**, increases in TAb and NAb levels were observed around 2 weeks to 1 month post the third booster shots (TP7) compared to those at six months from the first doses (TP6). The peak levels of antibody responses before TP7 and those of TP7 were compared in these nine participants (**Figure 5**). The TAb levels (Peak TP1-6: 8.71[2.037-18.325] vs TP7 48.52[11.025-108.955], *p* = 0.0382, Wilcoxon test) were significantly higher after the booster shots (**Figure 5A**), and the NAb inhibitions increased but did not show statistical significance (Peak NAb TP1-6: 50.7 ± 23.29% vs TP7: 69.4 ± 28.42%, *p* = 0.1027, paired t-test) (**Figure 5B**). Further, we compared the antibody avidity. The antibody avidity was measured by the decrease rate in fluorescent signals (dR) when the immunocomplex of recombinant RBD and serum SARS-Cov-2 antibodies dissociated in PBST buffer. A slower decrease in fluorescent signals represented by a smaller dR indicated a stronger binding between SARS-Cov-2 antibodies and RBD, which was verified by a previous study. The antibodies after the third dose showed significantly smaller dR (Peak dR TP1-6: 0.00113 ± 0.0003718 vs TP7: 0.00047 ± 0.0001315, *p* = 0.0001, paired t-test) (**Figure 5C**). Hence, the antibodies had higher avidities after the third dose, indicating antibody affinity maturation. Besides, we also compared two similar time points, one month after the second shot (TP4) and one month after the third shot (TP7). As shown, the TAb levels after the booster shots were significantly higher than the second shots (**Figure 5D**), and the NAb inhibitions after the booster shots increased but did not show statistical significance (*p* = 0.1915) (**Figure 5E**). The antibodies after the booster doses showed significantly smaller dR (**Figure 5F**).

### Safety evaluation by hematology and lymphocyte analysis

**Figure 6:**
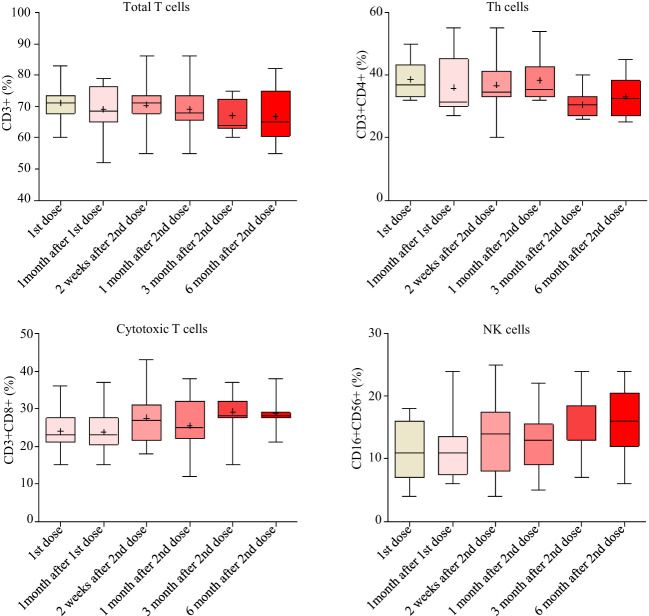
T cell responses measured by flow cytometry from baseline to 6 months after the second doses.

The white blood cells and the inflammatory biomarker CRP were monitored from TP1 to TP6. The counts of white blood cells, the counts and percentages of neutrophils, lymphocytes, eosinophils and basophils, as well as mean volumes and counts of platelet remained normal during the follow-up. CRP remained at low levels (median [IRQ], 1.6 [1.6-1.615] mg/L) during the study. The percentages of total T cells, cytotoxic T cells, T-helper cells and natural killer cells had very little variations during the study (**Figure 6**). No association was found between the peak antibody levels and the baseline CRP, hematology or lymphocyte measurements. However, we did not measure the antigen-specific T cell responses.

## Discussion

One of the most crucial methods for controlling and preventing COVID-19 is vaccination, and many platforms and technologies have been employed to create and use vaccines across diverse nations. The conventional inactivated viral vaccines are also efficacious for COVID-19, despite the fact that mRNA vaccines, which got their first-in-class licensures during this pandemic, are readily available worldwide. One of the inactivated viral vaccines that is frequently utilized in China is the BBIBP-CorV vaccination. The longitudinal investigations on the immune responses of immunized healthy volunteers offer crucial proof of the vaccine’s efficacy and perhaps even details on the length of protection offered by each dosage. In this study, we monitored the kinetics of the antibody responses in eighty-five healthcare workers who received the BBIBP-CorV vaccine over the course of about 10 months. Throughout the study time, they were all free of infection, hence this research offered information on vaccination in healthy individuals.

In this work, the Pylon assays were used to evaluate the antibody responses in three areas: total anti-SARS-Cov-2 antibody levels (TAb), surrogate neutralization antibody levels (NAb), and antibody avidities. At the beginning, every participant tested negative. Using Pylon TAb assays (29.4%) and Pylon NAb assays (11.8%), the seroconversion was detected as early as the time of the second doses, which were administered one month following the first doses. After the second dose, the positive rates grew even further, reaching 88.2% between two weeks and one month (**Table 2** and **Figure 1**). Although we did not begin collecting samples until one month after the baseline, it seemed quite possible that the seroconversion took place even earlier. The premarketing clinical trials of BBIBP-CorV showed seroconversion from 7 days after the first doses and higher positive rates (~100%). Another study in China (90.7%) (Zhang et al., 2021) and two studies outside China (81.5-83%) (Lijeskić et al., 2021; Petrović et al., 2022) showed similar positive rates to this study. The difference may result from the sample sizes and the methods to measure the antibody levels. In the participants of this study, the positive rates already decreased by 3 months after the second dose and further declines were observed around 6 months after the second dose. Similar to the positive rates, the TAb levels and the NAb levels showed the same trends with the peaks observed by one month after the second dose and significant declines by three months after the second dose (**Figure 2**). This trend was also found by other studies on BBIBP-CorV (Badano et al., 2022) and other vaccines including mRNA vaccines BNT162b2 (Lijeskić et al., 2021), showing drops in the antibody levels and neutralization ability by 3 months post vaccinations. We found the peak antibody levels were associated with age (Spearman’s of -0.674 and -0.7 for TAb and NAb vs age respectively, *p*<0.001), but were not associated with gender, BMI and hematology test results. The participants younger than 30 years had significantly higher TAb levels than the others (**Figure 4**). The association of age with humoral responses to the vaccines is consistent with other studies on BBIBP-CorV (Xia et al., 2021; Zhang et al., 2021) and other vaccines, e.g. BNT162b2 (Petrović et al., 2022) that showed slower seroconversion and lower antibody levels in the elderly. Zhang et al. also found those who failed in seroconversion had higher SAA levels and lower lymphocyte counts (Zhang et al., 2021), but that was not observed in the participants of our study.

Booster shots have been encouraged worldwide to enhance the humoral immunity that decayed months after the previous vaccination. Few studies are available on the booster shots of BBIBP-CorV, but early data showed that the booster shots of BBIBP-CorV and another inactivated virus vaccine (CoronaVac) increased antibody levels and neutralization ability on mutated variants and improved protection of hospitalization, severe cases and mortality (Ai et al., 2022; McMenamin et al., 2022). In this study, although only part of our study participants had booster shots, the paired comparison showed increases in the TAb levels (*p* = 0.0382, Wilcoxon test) and the NAb inhibition abilities (*p* = 0.1027, paired t-test). Furthermore, for the first time, the antibody avidities in healthy subjects after the vaccination of BBIBP-CorV were measured and significantly higher antibody avidities were observed after the booster shots (*p* = 0.0001, paired t-test). That indicated that the maturation of antibody affinity could take place in healthy subjects by multiple shots of BBIBP-CorV vaccines, similar to the antibody maturation in COVID-19 patients (Luo et al., 2021). Therefore, the booster shots not only improved the quantity of anti-SARS-CoV-2 Abs but also the quality, leading to more effectiveness in the prevention of infection and disease progression. This added evidence on promoting the use of booster doses in healthy populations.

It was noted that the study had several limitations. A small number of participants were included although a relatively long follow-up period was achieved. Also, due to the limitation of resources and facilities, we were not able to study the humoral responses to live viruses or different virus mutants. Finally, the quantitative results reported here would not be able to compare directly with other studies due to lack of the standardization among SARS-CoV-2 antibody assays in general. However, it should not affect the longitude analysis of antibody kinetics in this study.

## Conclusion

In conclusion, our findings showed that the two doses of the inactivated viral vaccine BBIBP-CorV may induce humoral immunity in healthy individuals, which degraded three months after immunization. The BBIBP-CorV booster shots increase both the quantity and quality of antibodies, providing proof that booster shots should be given to prolong the benefits of vaccination.

## Data availability statement

The original contributions presented in the study are included in the article. Further inquiries can be directed to the corresponding author.

## Ethics statement

The studies involving human participants were reviewed and approved by the medical ethics committee of Shanghai East Hospital. The patients/participants provided their written informed consent to participate in this study.

## Author contributions

JW, HM and XP analyzed and interpreted the data. WG was a major contribution in writing the manuscript. All authors contributed to the article and approved the submitted version.
